# Interventions to support postpartum health and well-being of parents with infants in neonatal intensive care units: a scoping review

**DOI:** 10.3389/frhs.2026.1845396

**Published:** 2026-07-15

**Authors:** S. Verbiest, K. Bryant, E. McClain, E. Morris, E. Cox, S. T. Wright, I. Asiodu, A. Stuebe

**Affiliations:** 1 School of Social Work, University of North Carolina at Chapel Hill, Chapel Hill, NC, United States; 2 Collaborative for Maternal and Infant Health, Department of OB/GYN, School of Medicine, University of North Carolina at Chapel Hill, Chapel Hill, NC, United States; 3 Division of Neonatal-Perinatal Medicine, Department of Pediatrics, School of Medicine, University of North Carolina at Chapel Hill, Chapel Hill, NC, United States; 4 Health Sciences Library, University of North Carolina Chapel Hill, Chapel Hill, NC, United States; 5 Motivating Interdisciplinary Lactation Knowledge (MILK) Research Lab, Department of Family Health Care Nursing, School of Nursing, University of California San Francisco, San Francisco, CA, United States

**Keywords:** family-centered care, maternal health, neonatal intensive care units, NICU mental health, NICU parents health, postpartum

## Abstract

The postpartum period is particularly vulnerable for families with an infant in the NICU, where stress, separation, and parental health needs are common. This scoping review, conducted in accordance with PRISMA-ScR guidelines, identifies interventions supporting the postpartum health and well-being of NICU parents. Electronic searches included articles from January 1, 2014, to April 18, 2024, published in English that included more than 20 participants from high-income countries. After duplicate removal, 4,799 abstracts were screened, and 87 articles met inclusion criteria via dual review. Interventions were grouped into six categories: postpartum physical and social care; mental health care; NICU design and technology; parent-infant interaction enhancement; family-centered care; and infant care and development education. Family-centered care, parent-infant interaction enhancement, and infant care education improved parental well-being. However, gaps remain regarding interventions focused on improving parental physical and mental health, signaling the need for studies addressing postpartum health in the NICU.

## Introduction

The postpartum period is a very sensitive and transformative period in the life course of the mother, infant, and family. Women experience rapid physical changes, post-delivery recovery, emotional and psychological adjustments, and hormonal shifts (1). These changes are often paired with limited sleep, return to paid employment, and significant caregiving responsibilities for a newborn and often other family members (1). Maternal/paternal-infant bonding and identity formation are other critical growth points. The postpartum experience overall has largely been neglected, under-resourced, and insufficiently funded in the United States (US) (2, 3). As a result, many families lack the comprehensive support and care needed to achieve optimal health and well-being after birth. These challenges are exacerbated when a family's newborn is hospitalized.

Mothers and infants in the early months of life are considered by evolutionary pediatrics to be interdependent, both physiologically and behaviorally (4). Close physical proximity, feeding, and caregiving of the infant by the mother increases prolactin, stimulating milk initiation and supply, and oxytocin, promoting attachment and sleep, while decreasing maternal and infant cortisol (4). In addition to recovery from childbirth and shifting hormones, this “fourth trimester” is also an intense time of role transition and expanding responsibilities for birthing and co-parents (2). During an admission to the neonatal intensive care unit (NICU), maternal/paternal - infant bonding, caregiving, and parental identity formation are significantly disrupted (5, 6). Additionally, anxiety, trauma, and stress are often high due to the infant's health status, the environment, and physical separation from the infant (5). NICU staff and parents are focused on the neonate, which often leaves maternal well-being as an afterthought. This neglect has serious potential consequences, as mothers with infants receiving intensive care are more likely to have significant chronic health conditions and unmet social, emotional, mental, and physical health needs than other women (7). The Care4Moms study found that over half of women with infants in the NICU were recovering from cesarean birth, 7.5% experienced severe maternal morbidity, and 6.6% required a blood transfusion (7, 8). While infants in the NICU are receiving state-of-the-art treatment, mothers often lack access to basic care. In the Care4Moms study, women with hypertensive disorders whose infants were hospitalized in a tertiary care setting were no more likely to receive a recommended blood pressure check at 7–10 days postpartum than women whose infants had been discharged from the NICU (7). Postpartum mothers report unmet needs for primary care, family planning, lactation support, mental health screening and treatment, and tobacco cessation programs (9).

Postpartum women with hospitalized infants have interrupted experiences with lactation and challenging healing and recovery experiences that put them and their baby at risk for ongoing poor outcomes (10). NICU families often report increased feelings of stress, anxiety, guilt, and shame (5), and they report experiencing high levels of depression and birth trauma (11–13). Harris and Gibbs, et al., found that mothers of preterm infants have higher rates of maternal mental health challenges than other mothers (14). Post traumatic stress symptoms affect 23% of NICU moms potentially increasing to 40% by 14 months post-delivery (14). Witt, et al.'s, work describes the stressors shared by Black and Hispanic mothers with NICU babies, including postpartum medical complications, stressful life events, poor provider communication, lack of social support, financial concerns, and work challenges (15). Research has shown that from a population perspective, household income drops to the lowest lifetime level and poverty rises to the highest in the months before childbirth through the child's first year, a situation likely made more difficult for families managing the additional and significant expenses of having a hospitalized infant (16, 17).

NICUs are designed to provide critical care to infants and often do not accommodate the needs of postpartum women and families. Seven editions of standards for newborn ICU design were published without addressing the need for “sufficient furnishing to allow a parent to stay seated, reclining, or fully recumbent at the bedside” (18, 19). NICU families describe challenges with basic practical needs, such as NICU visitor restrooms without sanitary products, going without meals to avoid leaving the infant's bedside, not taking pain medication, and pulling over on the side of the road to sleep because there was no place to rest while visiting the NICU (9). Postpartum women whose infants were critically ill or unstable were generally unwilling to seek care away from their infant's bedside, even when faced with their own serious health needs (9).

In response to these postpartum concerns, our study aims were to 1) identify interventions that address the needs of parents of infants hospitalized in a NICU and 2) understand the reported effects of these interventions on improving their health and well-being. These aims are exploratory and require a review of current evidence-based practices. As scoping reviews seek to systematically understand the breadth of evidence on a topic within a particular context and identify key related factors, this approach best fit our purpose (15). Recognizing the dyadic nature of the parent/infant relationship, this scoping review is part of a multi-faceted project—Care for NICU Families—that is working to describe model policies and best practices to improve clinical care, community supports, and access to services for postpartum families with infants in the NICU. We have used “parent” in our work to be inclusive of fathers, partners, and other caregivers who are an essential part of the infant's life, provide support to the dyad, and experience distress, yet are often overlooked. At the same time, we would like to highlight that the majority of the studies focused on mothers. The results of this review will combine with findings from community listening sessions, an environmental scan, and the expertise of professional and lived-experience advisory groups to offer recommendations to improve NICU postpartum care practices. Given the significant bio-psycho-social needs of postpartum parents with hospitalized infants, the results of this review and our recommendations will hopefully guide future interventions and studies to improve care for families at this sensitive period of development.

## Methods

In reviewing interventions, we considered their reported effects on parental physical and emotional health during the postpartum period. We followed the Preferred Reporting Items for Systematic reviews and Meta-Analyses extension for Scoping Reviews (PRISMA-ScR) guidelines for our work (20). A protocol was not registered in advance of completing the scoping review.

A trained clinical health sciences librarian (S.T.W.) performed our comprehensive electronic search of publications using the following databases: PubMed, Cumulative Index to Nursing and Allied Health Literature via EBSCO, EMBASE via Elsevier, Scopus, and PsycInfo via EBSCO. Our search was restricted to papers published in English. All database results were collected from January 1, 2014, through April 18, 2024. Search terms were used to retrieve articles addressing the three main concepts of the search strategy: (1) parents/family; (2) Neonatal Intensive Care (NICU); and (3) support, outcomes, or program development. The exact search strategy used in each electronic database is reported in Supplementary Appendix. The search strategy was conducted in PubMed using keyword and MeSH combinations. All databases used a combination of extensive keywords and, if available, database-specific controlled vocabulary. Results were downloaded to EndNote, and duplicates were removed. All references were uploaded to Covidence Systematic Review software, a web-based tool designed to facilitate and track each step of the abstraction and review process (21).

Our inclusion criteria for the scoping review are described in Table 1. Literature reviews, quality improvement projects, as well as quantitative and qualitative studies that met the minimum sample size were eligible for inclusion. We did not include commentaries, case studies, opinion papers, society position papers, conference abstracts, or book chapters.

**Table 1:** Inclusion criteria and rationale (21).

Inclusion criteria	Rationale
**Publication**: Dates from January 1, 2014, through April 18, 2024.	The Affordable Care Act in the United States went into effect January 2014, leading to major improvements in access to health care. Systematic reviews that included articles published before this data were excluded.
**Language of publication:** English	Resource and staffing limitations.
**Population:** Mothers/Parents/Caregivers/Fathers of infants hospitalized in a neonatal intensive care unit.	Parents of infants who were stillborn were not included. Studies that focused only on infants were excluded, as were studies that only focused on providers and NICU staff.
**Interventions:** Best practices, policies, interventions, and services targeting the health and well-being of parents while the infant is hospitalized and in the transition home, up to one year postpartum.	Focused on studies that did more than just describe parental needs during and after their neonate's hospitalization but addressed a component of parental well-being. Study protocols were excluded.
**Context:** High-income countries as per World Bank Group Country Classifications by Income Level for FY24	We anticipated that NICUs in high income countries would have similar access to health care provider training, neonatal care standards, technology, and medications across countries.
**Outcomes**: Parental physical and mental health indicators, care utilization, diagnoses; social determinant of health indicators; disparities in parental outcomes; Neonatal length-of-stay and readmission.	These outcomes were chosen as ones that contribute significantly to parental physical, mental, emotional, and financial well-being.
**Study Type**: Studies with >20 parent participants; observational studies; intervention studies; systematic reviews; qualitative studies; descriptive reports of community interventions; and program description/evaluation.	Included study types that provided detailed information about interventions and their effect on the mental and physical well-being of NICU parents. Commentaries, case reports, studies with <20 parent participants, book reviews, and animal studies were excluded.

We utilized the Covidence platform to support our review process. At the beginning of each phase of the review, the team reviewed a subset of articles, then confirmed shared understanding, and documented inclusion/exclusion decisions. Two team members reviewed each abstract and full manuscript. At least two team members were involved in resolving conflicts about article inclusion. An extraction data collection form was designed and iterated in Covidence. Full article extraction was completed by one team member and reviewed by a second team member. Items included in the data extraction were study ID, author, title, country(ies), setting, study aim, study design, publication year, participant description, total number of participants, type of intervention, outcomes measured, and outcome summary. Team members extracted additional details about the intervention and the measurement tools used for the included studies. Three team members grouped the extracted articles into six categories. A critical appraisal of the individual sources of evidence was not conducted.

## Results

**Figure 1:**
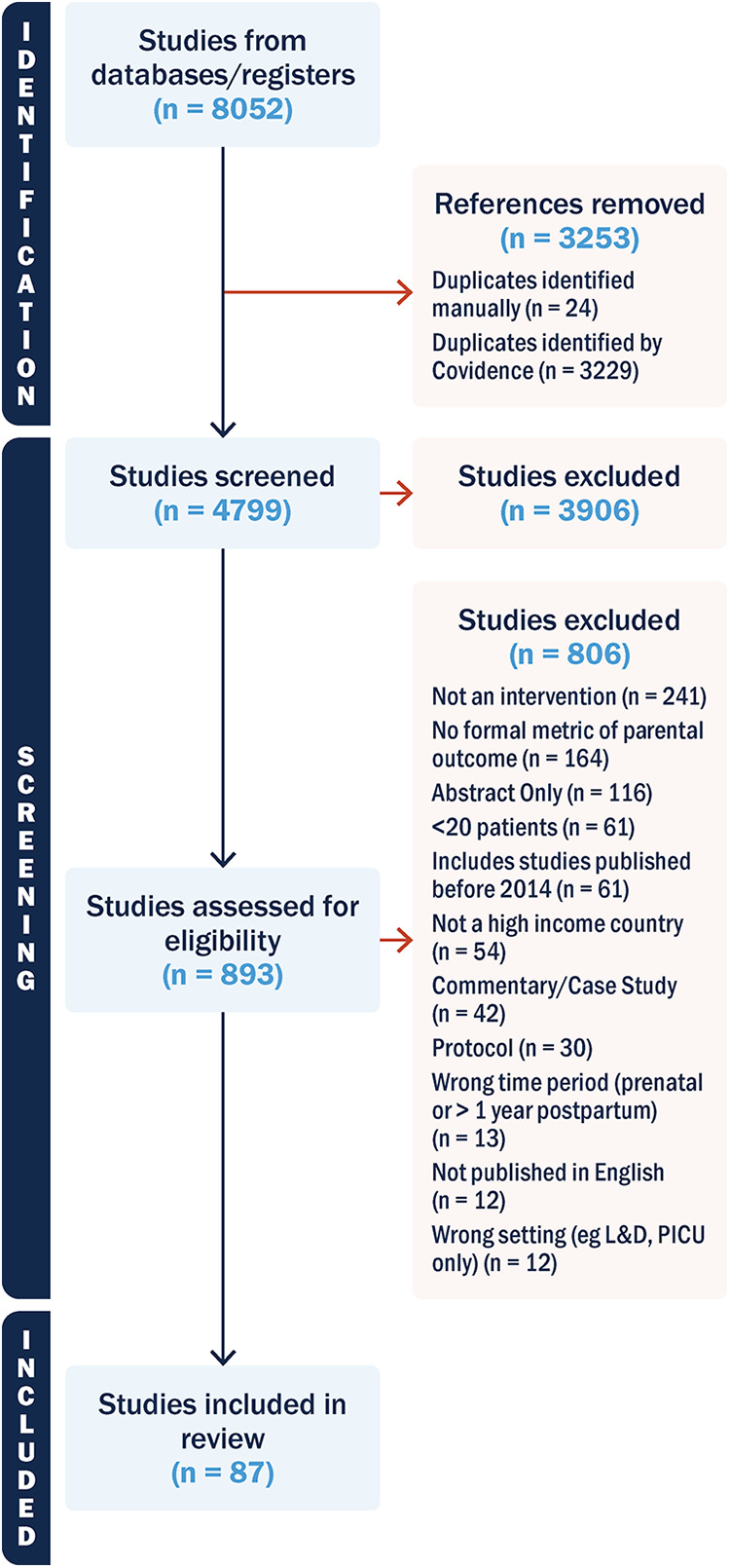
Evidence selection (21).

Our initial search identified 8052 articles. Once we eliminated duplicate entries, we reviewed 4,799 abstracts. We then moved to full manuscript review to identify the final articles included in this review. Figure 1 describes the selection of our sources of evidence.

We reviewed and excluded many papers that defined parental postpartum challenges but did not test programs or interventions to address identified problems. Many reviewed abstracts did not include any parental outcomes or measures. We initially included parental satisfaction on the advice of our lived experience advisory group; however, most of these studies only focused on infant healthcare related interventions and assessed parental satisfaction with the infant-focused intervention. While parent satisfaction with infant care is critical and contributes to parental well-being, studies in which satisfaction was the only parent outcome measured were excluded.

We included manuscripts that described interventions across 24 high-income countries. Most studies focused on maternal needs—very few included paternal health or partner/other caregiver interventions. When “parents” or “caregiver” terms were used, they largely referred to mothers. In reporting, we refer to the study population using the same terms as the authors. Infants with congenital anomalies and/or life-threatening conditions were routinely excluded from research studies, thus their parents' needs were largely excluded.

Supplementary Table 3 provides details of the 87 papers that were included in our review. The studies are grouped into the following intervention-related categories: Postpartum Physical and Social Care; Mental Health Care; NICU Design and Technology; Parent-Infant Interaction Enhancement; Family-Centered Care; and Infant Care and Development Education. While some manuscripts potentially fit into two groups, our purpose was to facilitate discussion rather than create definitive categories. The studies are described in greater detail below.

### Postpartum physical and social care

While a primary interest for our review, we only identified seven studies that addressed parental physical or social health issues. Dunlop, Logue, et al., offered enhanced intervention and counseling sessions to support postpartum visit attendance, folic acid daily consumption, and correct contraceptive use, as well as providing information and support for changing behaviors around alcohol consumption and birth spacing (22). Their results suggested that implementing maternal risk assessment and intervention in the NICU setting could be beneficial (22). Verbiest, McClain, et al., documented health care encounters provided to mothers of NICU infants by a certified nurse midwife (23). The study identified that this population has a myriad of health care needs, many of which can be addressed by or near the infant's bedside in less than 30 min (23). Mothers were open to receiving health care services in the NICU, particularly from a provider with the capacity to write prescriptions (23).

Two studies focused on providing support for lactating mothers. Massa, Ramireddy, et al., trialed an intervention that integrated mindfulness-based meditation while expressing breastmilk which did not demonstrate a clear effect on mental health or breastfeeding, but did suggest a potential benefit to using a meditation app while pumping (24). Ericson, Eriksson, et al., explored proactive daily telephone calls to offer support to mothers who were providing breastmilk to their babies (25). While there were no statistical differences, the mothers in the intervention group reported lower parental stress and felt more supported than those in the control group (25). We identified many papers focusing on breastmilk for infants in the NICU, but they were excluded as descriptive, small sample size, or focusing on lactation and infant outcomes, not the physical or mental health of the person providing breastmilk.

Two studies addressed reducing tobacco smoke exposure through (1) motivational interviewing and financial incentives and (2) motivational advice and nicotine replacement therapy provision compared to Quitline referrals (26, 27). The studies suggested that parents are open to behavior change, and that offering services, incentives, and nicotine replacement therapy are acceptable, but more research is needed (26, 27). Finally, Cordova-Ramos, Jain, et al., demonstrated that it is feasible and acceptable to implement a systematic risk screening and referral intervention to address unmet social needs in the NICU setting, which in turn improves parental well-being (28).

### Mental health care

All 23 studies in this category emphasized the high risk for perinatal depression and anxiety among parents of infants in the NICU. While anxiety and depression decreased over time, the need for services was notable. Studies that sought to address parental mental health focused on screening and referral, including provision of in-unit services (7 studies) and interventions to actively address mental health challenges throughstress management, music therapy, and mindfulness or journaling (16 studies). Overall, these programs demonstrated reductions in depression, anxiety, and stress for families.

Seven studies focused on screening for postpartum anxiety and depression with referral to services as needed (11, 29–34). While each study had a different approach, they all demonstrated the feasibility of implementing screening and referral programs in NICUs. Screening approaches included (1) screening completed by bedside or unit nurses, (2) paper copies of screening tools left for parents to complete at the baby's bedside, (3) utilizing health profession students to conduct screenings, and (4) parents receiving a 30 min consultation that included a complete screening and introduction to NICU mental health services (11, 29–34).

All screening programs had mechanisms for follow-up and referral for people who had symptoms of anxiety and/or depression. These referral mechanisms are critical, as screening is essential but not sufficient unless it is connected to mental health care. Programs that proactively brought mental health services to parents were more successful in engaging them in treatment than those where mothers only received referrals to providers. Several studies did not screen mothers until 2 weeks postpartum to avoid the early “postpartum blues” that occur with hormonal fluctuations, which may have led to missing women with mental health needs whose infants were discharged during that timeframe.

Seven studies focused on interventions aimed at reducing stress and anxiety through mindfulness, stress management, and journaling (35–41). The interventions varied in duration and mode of delivery; however, they all sought to provide parents with skills and tools to practice mindfulness or write out their feelings (35–41). Overall, parents reported using mindfulness techniques and journaling, signaling that these were useful tools to help them cope with the stress of a NICU stay (35–41). The interventions were generally low-cost and feasible to implement (35–41). Many of the intervention groups were small, but the reported results were promising.

Three RCT studies examined the impact of dyadic music therapy on parental mental health. Kobus and Diezel (recorded music) and Kraft and Jaschke (live performed music) focused specifically on mothers and found reductions in anxiety and distress (42, 43). Menke and Hass (live performed music) included both parents and caregivers and reported promising results for infant development and parental wellbeing, although they called for further study to determine if family-centered music therapy should be part of routine NICU care (44).

There were a variety of measurement tools used to assess parental anxiety, depression, and mental health, which makes it difficult to compare outcomes across studies. The most used tools include the Edinburgh Postnatal Depression Scale (EPDS) (45), the State-Trait-Anxiety Inventory (STAI) (46), the Patient Health Questionnaire-9 (PHQ-9) (47), and the Parental Stressor Scale—NICU (PSS NICU) (48).

### NICU design and technology

Our review included 10 articles focused on NICU layout and the use of technology as related to parental postpartum well-being. Two studies compared parental experiences in a Single-Family Room (SFR) setting with those in an Open Bay (OB) setting. Campbell-Yeo, Kim, et al., studied whether the SFR or OB setting resulted in differences in parental presence, parental involvement in caring for their infant, and maternal well-being (49). The study found that mothers were present for longer periods of time and spent more time expressing breastmilk in the SFR setting (49). Fathers were present for and provided more infant care in the SFR setting (49). In the Open Bay setting, parents held infants more when clothed (not skin-to-skin), and mothers spent more time singing or reading to their infant compared to those in the SFR setting (49). There were no significant differences between the two settings' parental well-being measures (49). Tandberg, Flacking, et al., studied how NICU design affected parental presence, depression, stress, anxiety, and maternal attachment (50). The SFR and OB unit in this study were in two different hospitals, and the supports provided to parents were different, with the SFR setting's policies not limiting parental presence and providing more physical supports (e.g., meals and private sleep space with the infant) (50). In the SFR setting, mothers had lower EPDS scores, parents had lower STAI scores, lower Short Form Y (STAI SF) scores while in the hospital, and lower PSS-NICU scores (50). Parents were present for much longer periods of time in the SFR setting (50). There was not a significant difference in parenting stress or maternal attachment scores (50).

Neri, Genova, et al., assessed parental stress and feelings about the NICU space before and after a mural was painted on a NICU wall (51). There were no significant differences in parental distress between the two groups; however, parents in the NICU after the wall was painted rated the NICU space more positively than the other parents (51). Andersen, Holm, et al., studied the introduction of neonatal homecare in Denmark (52). This study used an interrupted time series analysis to measure the effect of neonatal homecare, which allows the mother-infant dyad to be at home for the final weeks of NICU admission, on severe postpartum depression in mothers (52). The team used national registry data for their analysis and found that severe postpartum depression was reduced following the introduction of neonatal homecare; however, this was not seen in all populations (52).

Ahn and Jo studied the impact of an intervention to limit the spread of COVID-19 on parental well-being (53). The non-contact visit program consisted of a video call that included introductions, development activities including vitals, nursing care, coping, baby care, and time for communication between the mother and their baby (e.g., singing) and follow up parent education in a file delivered electronically (53). Parents in the intervention group perceived an increase in nurse support; however, there was not a statistically significant difference between the two groups in parental stress (53).

Parental use of webcams to view their infant remotely was the focus of four studies included in this review. Reimer, Mause, et al., interviewed parents about what they expected or experienced related to webcams (54). Parents shared having increased feelings of closeness to their infant, feeling reassured, improved ability to express breastmilk, and good relationships with providers (54). Interviewed parents also noted negative effects on relationships between parents and providers, concerns about privacy, a negative impact on visitation, and increased stress (54). In a second study, Kerr, King, et al., found through parent interviews that parents' experiences with the webcam were generally positive (55). Parents noted feeling more connected, more emotionally stable, and improved breastmilk production (55). This study allowed video to be shared with extended family (55). Parents also noted increased anxiety, needing to call to understand what was happening, not knowing why the camera would be turned off for long periods of time, and seeing procedures they did not want to see if the camera remained on (55). Professionals were also interviewed in this study and felt that webcams were generally positive for parents (55). They noted concerns about forgetting to turn off the camera during an emergency or parents seeing procedures (55).

Kubicka, Zahr, et al., evaluated the experiences of both parents and nurses when webcams were universally unavailable (September 2018–March 2019) and were universally available (March–Augest 2019) to parents of infants admitted to a NICU (56). There was not a statistically significant difference in parent-reported stressors at baseline between the parents with and without webcam access (56). The majority of parents with webcam access felt that watching their baby via webcam made them feel better and reassured them about the care their baby was receiving (56). Parents without webcam access had significantly higher stress measure scores on the PSS: NICU than parents with webcam access (56). Legge, Middleton, et al., assessed parent and staff experience before and after the implementation of NICU webcams (57). They found that once webcams were implemented, more parents skipped NICU visits to address other needs (e.g., care for other children), and fewer parents thought they would feel better if they visited for longer (57). Most parents were supportive of using the webcam, and there were no significant differences in the scores between the two groups on the Depression Anxiety Stress Scale (DASS-21), though there was a downward trend after implementation (57). Kirolos, Sutcliffe, et al., evaluated the use of a video diary intervention, allowing parents to see videos of moments that they were not present for in the NICU (58). Families indicated that the intervention had a positive effect on several aspects of their well-being, including stress and anxiety, sleep, breastmilk expression, feeling reassured, emotional closeness, and involvement in care (58).

### Infant care and development education

Our review included 10 articles focused on educating parents about their premature infants. Gibson, Williams, et al., studied the impact of the Babble app on parents' self-efficacy, distress, and feeling informed (59). While feeling informed reduced distress and increased feelings of self-efficacy, Babble app use was not significantly associated with improving parental well-being (59). Erdei, Forde, et al., conducted a pilot study to assess how the My Brigham Baby app affected parent discharge readiness, parenting competence, stress, and anxiety (60). The app included information about the experience in the NICU, links to external support, provider communication, and some medical information about the infant (60). They found a significant increase in discharge readiness, an increase in parenting competence and stress, and a decrease in parental anxiety (60). Collette, Feeley, et al., conducted a pilot study of a French language website about the NICU compared to an electronic pamphlet, assessing how the website affected parental psychological well-being (61). Individuals in the intervention group experienced a decrease in depressive symptoms, and all participants experienced reduced stress, though this effect was larger in the intervention group (61).

Hoffenkamp, Tooten, et al., studied the effect of Video Interaction Guidance (VIG) on parental bonding, sensitivity, mental health, and trauma (62). They found a significant difference in the bonding and detachment scores of parents who received VIG early in the NICU stay and significantly higher bonding scores, particularly for fathers and mothers who experienced trauma (62). There was no significant difference between the groups on mental health outcomes (62). Borghini, Habersaat, et al., studied the effect of a three-component intervention on maternal posttraumatic stress and the quality of interactions between the mother and infant (63). The intervention included joint infant observation by the parent, nurse, and therapist, an assessment of the infant using the Neonatal Behavioral Assessment Scale, and an interview with the mother using the Clinical Interview for Parents of High-Risk Infants, as well as videotaped interactions between the mother and infant that were followed by feedback for the mother (63). Mothers who received the intervention experienced a significant decrease in posttraumatic stress compared with mothers who did not receive the intervention, as well as significant improvements in infant interactions (63).

Cano Gimenez and Sanchez-Luna assessed the impact of a five-part intervention on stress and mental health (64). The intervention included orientation to the NICU setting and procedures, guidance, education, coping support, and planning support (64). While there were no significant differences between the two groups at admission, mothers in the intervention group had lower stress levels (64). Anxiety was significantly lower in the intervention group after 15 days in the NICU, and depression rates were significantly lower at discharge (64). Chen, Lee, et al., studied fathers receiving additional educational materials about caring for their infant, as well as the ability to engage with a nurse for guidance, education, support, and to ask questions (65). While there was no significant difference in paternal stress between the groups at baseline, fathers that received the intervention had lower stress rates, higher scores on supporting mothers, higher attachment scores, and significantly higher scores of fathering ability at discharge (65).

Ji and Shim assessed how a home visiting intervention with an experienced NICU nurse and a thorough infant physical examination, in addition to a standard community home visiting and support group participation, affected stress, competence, and coping (66). While the intervention did not result in a significant difference in parental stress or efficacy, there was a significant improvement in coping (66). Fratantoni, Soghier, et al., studied the difference between a standard group that received a care notebook to prepare for discharge and a group that received the notebook and had a peer navigator that supported them in identifying resources and communicating with providers and provided emotional support in the year after discharge (67). The study assessed parental stress, maternal self-efficacy, depression, anxiety, and infant outcomes (67). There were no differences in outcomes between the two groups during the study period; however, in the standard group, there were lower levels of stress three months after discharge (67). Mental health improved in both groups (67). Dahan, Bourque, et al., studied an intervention with weekly support meetings, with most parents finding them useful (68). Three major themes emerged about participating in the meetings including (1) reduced isolation and feeling part of a community, (2) feeling more hopeful and resilient, and (3) practical information for parents (68). The authors reported that parents expressed appreciation for hearing stories and sharing, the friendliness of the group, and the quality of the resource parents (68).

### Family-centered care

Our review included 17 articles that focused on an aspect of family-centered care, including policies around parental presence in the NICU and implementation of different models of family-centered care. Those models included Family Integrated Care (FICare, 9 articles) (69–77), Family-Centered Care (FCC, 3 articles) (78–80), Close Collaboration with Parents (2 articles) (81, 82), and Creating Opportunities for Parent Empowerment (COPE, 1 article) (83). Six of the FICare articles were based on two studies: one in the Netherlands, and the other in Australia, Canada, and New Zealand.

The studies largely focused on the outcomes of implementing family-centered care models or policies on parental stress, depression, anxiety, and post-traumatic stress. The most common measurement tools used were the Parental Stressor Scale: NICU (PSS:NICU) (48), the State Trait Anxiety Inventory (STAI) (46), and the Edinburgh Postnatal Depression Scale (EPDS) (45). Across every study, implementation of family-centered care models and policies resulted in significantly lower stress, anxiety, and/or depression scores and symptoms among parents of infants in the NICU. When fathers were included in the studies, they also exhibited significantly lower stress, anxiety, and depression scores and symptoms following the implementation of family-centered models and policies. Additionally, in two studies that followed families for an extended period of time [18 months—McLean, et al. (76), and 2 years corrected age—Ahlqvist-Björkroth, et al. (82)], mothers whose infants were cared for in NICUs that implemented family-centered care models continued to exhibit significantly lower cortisol levels and depression scores when compared to the mothers of infants cared for in a NICU that did not implement family-centered care models (76, 82). Franck, Gay, et al, did not see an overall significant difference in PTSD or depression scores between groups receiving family-centered care (FCC) and mobile-enhanced Family Integrated Care (mFICare), but the authors did report an effect among mothers who had higher stress scores at baseline—the mFICare intervention was more effective than standard FCC at preventing clinically significant symptoms of PTSD in those mothers at four months post-discharge (77).

### Parent-infant interaction enhancement

There were 20 articles that focused on the reported effects on parents of interventions to enhance parent-infant interaction and contact, including skin-to-skin care (SSC, 5 articles) (84–88), kangaroo mother care (KMC, 9 articles) (89–97), Supporting and Enhancing NICU Sensory Experiences (SENSE, 2 articles) (98, 99), and infant massage (3 articles) (92, 100, 101). Kangaroo mother care, as defined by the World Health Organization (WHO), is the care of preterm and low birth weight infants through prolonged (at least 8 h per day) skin-to-skin contact, exclusive breastfeeding, and within a health system, the timely discharge to a lower level of care or home, as appropriate, with continued skin-to-skin contact and exclusive breastfeeding encouraged (102). It was not clear in every article that used the term KMC that they were strictly adhering to all of the WHO guidelines; the term used by the authors is the one used in this paper. The general consensus among the articles was that KMC and skin-to-skin contact lowers signs and symptoms of stress and anxiety in parents. Seven articles found statistically significant declines in stress and anxiety due to SSC/KMC (85, 88, 89, 92, 94–96). Three articles reported significant drops in maternal and/or parental cortisol levels during SSC/KMC (85, 88, 89), one article reported significant declines in parental blood pressure and heart rate (86), and another reported significantly higher levels of parental oxytocin (88). While many studies excluded parents of infants with congenital anomalies, one study explicitly focused on the implementation of SSC among dyads where the infant had congenital heart disease (85). Lisanti, et al., found that SSC before and after neonatal cardiac surgery resulted in significant reductions in mothers' anxiety and stress scores and salivary cortisol (85).

Three articles compared enhancements to SSC/KMC to routine SSC/KMC, including the addition of body wraps (84), mindfulness for mothers (93), and music therapist-guided singing for mothers (91). Freccero, et al., found that the addition of body wraps did not result in any statistically significant differences in parental stress scores or confidence in carrying out SSC, adverse events, or length of SSC sessions, when compared to typical SSC (84). Conversely, the addition of music therapist-guided singing/humming and the addition of mindfulness for mothers conducting KMC both resulted in significant reductions in stress and anxiety when compared to mothers who engaged in typical KMC (91, 93).

Six articles reported on parental outcomes of implementing multisensory infant care interventions, including the SENSE program (98, 99), Auditory-Tactile-Visual-Vestibular (ATVV) intervention (92), Family Nurture Intervention (FNI) (103), and infant massage interventions (100, 101). Pineda, et al., found that mothers who implemented the SENSE intervention had significantly higher maternal confidence scores (99); while Richter, et al., found that parents who completed most of the SENSE interventions for their infant had significantly lower stress and anxiety scores than parents who allowed staff to deliver the majority of SENSE intervention sessions (98). Welch, et al., similarly found that mothers who engaged in the multisensory calming activities of the Family Nurture Intervention (FNI) had significantly lower depression and anxiety scores at four months corrected infant age and engaged in more SSC sessions for a longer duration than the control group (103). This significant effect on depression and anxiety for FNI mothers held even when controlling for SSC sessions (103).

In the articles that reported on the parental outcomes of infant massage, McCarty, et al., found that mothers who engaged in infant massage had significantly lower salivary cortisol levels post-massage session (101), while McCarty, et al., also found that parents who engaged in infant massage had significantly lower anxiety and depression scores and higher self-reported parenting competence scores than parents who did not engage in infant massage (100). Similarly, Holditch-Davis, et al., reported that mothers who engaged in any infant massage, including ATVV, had a significantly more rapid decline in depression scores than mothers who did not engage in infant massage (92).

## Discussion

Infant care and feeding, along with parent-infant bonding, are core components of the postpartum period. The dyadic interplay underscores the deep connection between parents and their newborn. This developmental period is disrupted by a NICU stay, affecting both infants and parents. Neonatal nurses play an important role in parental education, skin-to-skin care, communication, and support. The studies we reviewed incorporated NICU nurses in a number of ways, including in the role of delivering interventions and receiving interventions toward improving patient care. We identified many studies that demonstrate that when parents are given opportunities to touch, hold, learn about, and care for their infant, their feelings of anxiety, depression, and stress are reduced. Likewise, when there is open communication between providers and parents, parental outcomes are improved. Family-centered care clearly emerged in our review as foundational to the health and well-being of both parents and infants. Unfortunately, family-centered care is not offered consistently in NICUs, and neonatal care providers vary in their ability to communicate and connect with families.

Parental mental health was a major theme of descriptive studies that we excluded as well as for the interventions we included. Many studies utilized the PSS:NICU scale as their main assessment of parental well-being. While this is a helpful tool, since it specifically asks about the stress caused by the NICU environment and medically fragile infant, it is inadequate in being able to fully assess parental mental health and trauma. Studies that paired this tool with the EPDS or STAI were able to more completely describe the parental experience and assess the effect of the intervention on their well-being.

We found only two interventions that sought to address the physical health needs of postpartum women, which include physical recovery from birth; chronic disease management; sleep and fatigue; and sexuality, contraception and birth spacing (104). These needs are particularly significant for mothers of medically fragile infants, who have higher rates of chronic diseases, complicated births, and acute care needs following birth (8). Co-locating maternal care services in the NICU has the potential to enable mothers to meet their acute needs without requiring them to leave their infant's bedside (7–9).

There were several interventions, such as a pilot study that provided Chinese medicine techniques to reduce anxiety and stress for NICU parents and providers, that seemed promising, but the sample sizes were very small. Overall, even most of the included studies had relatively small sample sizes, were pilot studies to prove the feasibility of the intervention, and/or did not provide data adequate to suggest introducing the intervention widely without further research. We identified papers that described the feasibility of screening for social determinants of health and building medico-legal partnerships for NICU parents but did not include parental outcomes or input. There were other interventions, such as webcams and NICU design, that could be further studied to see how they could be better used to support maternal sleep and postpartum recovery.

As we reviewed almost 900 complete manuscripts, we observed many studies that neglected to assess parental perspectives or only considered satisfaction with the intervention as it related to infant well-being. This was most striking in research on lactation, where the sole outcome of many studies was breastmilk output, with no assessment of how mothers felt about the intervention or approach. This was also true for studies on music therapy, skin-to-skin care, and parent education programs, among others, that were excluded as they only collected data on infant outcomes. We found very few interventions that were parent designed and/or co-designed, representing a missed opportunity. Likewise, there were very few studies that focused on interventions to address paternal needs, even though descriptive studies clearly delineated fathers' needs for support and services.

## Limitations

Our review was limited to high-income countries, and as such, we may have missed relevant research from other countries. For example, there were several promising studies in Iran related to spirituality support and NICU parent well-being. We only reviewed studies published between January 2014 and April 2024; therefore, this review does not include newly published work. We deployed a very wide variety of search terms to identify publications across a variety of topics and double-reviewed thousands of abstracts. However, it is possible that we excluded a relevant study in error. Increased specificity and language regarding the inclusion of fathers and other caregivers in the study and additional demographic information would have allowed us to report more refined results in some cases.

## Conclusion

This scoping review enabled us to identify interventions that address the needs of parents of infants hospitalized in a NICU and explore the reported outcomes of these interventions on improving the postpartum health and well-being of parents. Family-centered care is foundational for parental well-being. Parents' access to opportunities to hold and care for their infants, including kangaroo care, skin-to-skin care, and music therapy, can reduce NICU-related stress and foster parent-infant relationships and well-being. These activities normalize the NICU experience and offer mirror experiences they would have had if their baby were not hospitalized. Likewise, providing education on infant care and development can build parental self-efficacy and confidence, which can reduce stress. However, these interventions are inadequate for addressing the postpartum mental and physical needs of parents.

Descriptive and intervention studies, alike, underscore the significant and urgent mental health needs of parents of hospitalized infants. Maternal well-being has been linked to infant well-being and development; yet studies tend to focus largely on screening rather than on referrals or interventions to address concerns. Investment is needed in interventions co-designed with parents to address their unmet physical and emotional needs, particularly during the immediate postpartum period. There is an urgent need for large-scale, multicenter intervention studies and integrated postpartum care models embedded into NICU settings. Further, researchers must provide more explicit information about their population of focus, particularly their use of the word “parent” when their study largely focuses on mothers. Studies need to include father and partner-focused interventions and perspectives. Neonatal nurses play a key role in Family-Centered Care and support. Proposed interventions should both consider the importance of their role and identify additional health care team members with maternal health expertise to augment their capacity to meet the needs of complex families. Current research focuses on designing interventions to improve infant care and assessing parent satisfaction with those interventions, including most family-centered care studies. There is an unmet need for interventions designed by and for postpartum parents to address their healing and well-being that also assess the benefits of those interventions on hospitalized infants. Thousands of mother/parent-infant dyads experience the stress and challenge of a postpartum NICU stay—interventions that center their shared well-being and explore the inter-relationship of their needs and care are urgently needed.
