# Supplementary material for: Interventions to support postpartum health and well-being of parents with infants in neonatal intensive care units: a scoping review
Source: Front Health Serv. 2026 Jul 15;6:1845396. doi: 10.3389/frhs.2026.1845396 (PMC13416434; doi:10.3389/frhs.2026.1845396)
Supplement: Supplementary file 1 [file Table1.docx]

**Appendix A: Search Strategies**

**Pubmed**

**Searched April 18, 2024**

**2564 results**

("parenting"[MeSH Terms] OR "parents"[MeSH Terms] OR parent*[tiab] OR father*[tiab] OR mother*[tiab] OR maternal*[tiab] OR paternal*[tiab] OR family[tiab] OR families[tiab] OR family[mesh]) AND ("Intensive Care, Neonatal"[Mesh] OR "Intensive Care Units, Neonatal"[Mesh] OR nicu[tiab] OR nicus[tiab] OR ncu[tiab] OR "neonatal icu"[tiab] OR "neonatal icus"[tiab] OR "neonatal intensive care"[tiab] OR "neonatal critical care"[tiab]) AND (need*[tiab] OR intervention*[tiab] OR program*[tiab] OR initiative*[tiab] OR evidence[tiab] OR outcome*[tiab] OR program development[mesh] OR program evaluation[mesh]) AND (mindfulness[tiab] OR mindfulness[mesh] OR relaxation[tiab] OR participat*[tiab] OR stress*[tiab] OR round*[tiab] OR depress*[tiab] OR anxiety[tiab] OR "mental health"[tiab] OR psychosocial[tiab] OR support*[tiab] OR "Depression"[Mesh] OR "Depressive Disorder"[Mesh] OR "Depression, Postpartum"[Mesh] OR "Anxiety"[Mesh] OR "Social Support"[Mesh] OR "Teaching Rounds"[Mesh] OR "Stress, Psychological"[Mesh]) Filter: 2014 to present

**SCOPUS**

**Searched 4/18/2024**

**1332 results**

TITLE-ABS-KEY((parenting OR parent* OR father* OR mother* OR maternal* OR paternal* OR family OR families OR family) w/6 (nicu OR nicus OR ncu OR "neonatal icu" OR "neonatal icus" OR "neonatal intensive care" OR "neonatal critical care")) AND ((need* OR intervention* OR program* OR initiative* OR evidence OR outcome*) w/6 (mindfulness OR mindfulness OR relaxation OR participat* OR stress* OR round* OR depress* OR anxiety OR "mental health" OR psychosocial OR support* )) AND PUBYEAR > 2013 AND PUBYEAR < 2025 AND PUBYEAR > 2013 AND PUBYEAR < 2025

**EMBASE**

**Searched 4/18/2024**

**2451 results**

('parent'/exp OR 'child parent relation'/exp OR 'family'/exp OR parent*:ti,ab OR father*:ti,ab OR mother*:ti,ab OR maternal*:ti,ab OR paternal*:ti,ab OR family:ti,ab OR families:ti,ab) AND ('neonatal intensive care unit'/exp OR 'newborn intensive care'/exp OR nicu:ti,ab OR nicus:ti,ab OR ncu:ti,ab OR 'neonatal icu':ti,ab OR 'neonatal icus':ti,ab OR 'neonatal intensive care':ti,ab OR 'neonatal critical care':ti,ab) AND (need*:ti,ab OR intervention*:ti,ab OR program*:ti,ab OR initiative*:ti,ab OR evidence:ti,ab OR outcome*:ti,ab OR 'program development'/exp OR 'program effectiveness'/exp OR 'program evaluation'/exp) AND (mindfulness:ti,ab OR 'mindfulness'/exp OR relaxation:ti,ab OR participat*:ti,ab OR stress*:ti,ab OR round*:ti,ab OR depress*:ti,ab OR anxiety:ti,ab OR 'mental health':ti,ab OR psychosocial:ti,ab OR 'mindfulness-based stress reduction'/exp OR 'relaxation training'/exp OR 'physiological stress'/exp OR 'mental health care'/exp OR 'anxiety disorder'/exp OR 'postnatal depression'/exp OR 'teaching round'/exp)AND (2014:py OR 2015:py OR 2016:py OR 2017:py OR 2018:py OR 2019:py OR 2020:py OR 2021:py OR 2022:py OR 2023:py OR 2024:py)

**CINAHL**

**Searched 4/18/2024**

**1331 results**

((MH parenting+) OR (MH parents+) OR (MH family+) OR (TI parent* OR AB parent*) OR (TI father* OR AB father*) OR (TI mother* OR AB mother*) OR (TI maternal* OR AB maternal*) OR (TI paternal* OR AB paternal*) OR (TI family OR AB family) OR (TI families OR AB families)) AND ((MH "Intensive Care, Neonatal+") OR (MH "Intensive Care Units, Neonatal") OR (MH "Neonatal Intensive Care Nursing") OR (TI nicu OR AB nicu) OR (TI nicus OR AB nicus) OR (TI ncu OR AB ncu) OR (TI "neonatal icu" OR AB "neonatal icu") OR (TI "neonatal icus" OR AB "neonatal icus") OR (TI "neonatal intensive care" OR AB "neonatal intensive care") OR (TI "neonatal critical care" OR AB "neonatal critical care")) AND ((TI need* OR AB need*) OR (TI intervention* OR AB intervention*) OR (TI program* OR AB program*) OR (TI initiative* OR AB initiative*) OR (TI evidence OR AB evidence) OR (TI outcome* OR AB outcome*) OR (MH "psychosocial intervention") OR (MH "Program Development") OR (MH "Program Implementation") OR (MH "Program Planning") OR (MH "Program Evaluation") OR (MH "Hospital Programs")) AND ((TI mindfulness OR AB mindfulness) OR (TI relaxation OR AB relaxation) OR (TI participat* OR AB participat*) OR (TI stress* OR AB stress*) OR (TI round* OR AB round*) OR (TI depress* OR AB depress*) OR (TI anxiety OR AB anxiety) OR (TI "mental health" OR AB "mental health") OR (TI psychosocial OR AB psychosocial) OR (MH "Mental Health") OR (MH "Mental Health Counseling") OR (MH "Mental Health Services+") OR (MH "Anxiety+") OR (MH "Anxiety Disorders+") OR (MH "Depression+") OR (MH "Mindfulness") OR (MH "Mind Body Techniques") OR (MH "Self-Compassion") OR (MH "Patient Rounds") OR (MH "Depression, Postpartum") OR (MH "Postpartum Nursing") OR (MH "Postnatal Care")) **Limiters** - Publication Date: 20140101-20241231 and Academic Journals

**PsycInfo**

**Searched 4/18/2024**

**375 results**

((DE "Parent Child Relations" OR DE "Father Child Relations" OR DE "Mother Child Relations" OR DE "Parental Attitudes" OR DE "Parents" OR DE "Adoptive Parents" OR DE "Expectant Parents" OR DE "Fathers" OR DE "Foster Parents" OR DE "Homosexual Parents" OR DE "Mothers" OR DE "Parental Characteristics" OR DE "Single Parents" OR DE "Stepparents" OR DE "Surrogate Parents (Humans)" OR DE "Family" OR DE "Biological Family" OR DE "Dual Careers" OR DE "Dysfunctional Family" OR DE "Extended Family" OR DE "Family Background" OR DE "Family History" OR DE "Family Members" OR DE "Family of Origin" OR DE "Family Relations" OR DE "Family Resemblance" OR DE "Family Structure" OR DE "Family Work Relationship" OR DE "Interethnic Family" OR DE "Interracial Family" OR DE "Marriage" OR DE "Military Families" OR DE "Nepotism" OR DE "Nuclear Family" OR DE "Offspring" OR DE "Stepfamily") OR (TI parent* OR AB parent*) OR (TI father* OR AB father*) OR (TI mother* OR AB mother*) OR (TI maternal* OR AB maternal*) OR (TI paternal* OR AB paternal*) OR (TI family OR AB family) OR (TI families OR AB families)) AND ((DE "Neonatal Intensive Care") OR (TI nicu OR AB nicu) OR (TI nicus OR AB nicus) OR (TI ncu OR AB ncu) OR (TI "neonatal icu" OR AB "neonatal icu") OR (TI "neonatal icus" OR AB "neonatal icus") OR (TI "neonatal intensive care" OR AB "neonatal intensive care") OR (TI "neonatal critical care" OR AB "neonatal critical care")) AND ((TI need* OR AB need*) OR (TI intervention* OR AB intervention*) OR (TI program* OR AB program*) OR (TI initiative* OR AB initiative*) OR (TI evidence OR AB evidence) OR (TI outcome* OR AB outcome*) OR (MH "program development+") OR (MH "program evaluation+")) AND ((TI mindfulness OR AB mindfulness) OR (MH mindfulness+) OR (TI relaxation OR AB relaxation) OR (TI participat* OR AB participat*) OR (TI stress* OR AB stress*) OR (TI round* OR AB round*) OR (TI depress* OR AB depress*) OR (TI anxiety OR AB anxiety) OR (TI "mental health" OR AB "mental health") OR (TI psychosocial OR AB psychosocial) OR (DE "Depression (Emotion)" OR DE "Depression Screening")) OR (DE "Postpartum Depression")) AND (DE "Anxiety" OR DE "Anxiety Disorders" OR DE "Anxiety Management")) AND (DE "Stress" OR DE "Stress and Coping Measures" OR DE "Stress Management")) **Limiters** - Publication Year: 2014-2024 and Academic Journals
