# Supplementary material for: Interventions to support postpartum health and well-being of parents with infants in neonatal intensive care units: a scoping review
Source: Front Health Serv. 2026 Jul 15;6:1845396. doi: 10.3389/frhs.2026.1845396 (PMC13416434; doi:10.3389/frhs.2026.1845396)
Supplement: Supplementary file 2 [file Table2.docx]

**Abbreviations:**

**Measurement Tools**

| **Tool** | **Abbreviation** |
| --- | --- |
| Acceptability of Intervention Measure | AIM |
| Affective Quality Attributed to Place | QAL |
| Beck Anxiety Inventory | BAI |
| Beck Depression Inventory | BDI |
| Beck Depression Inventory-II | BDI-II |
| Behavioral Inhibition System and Behavioral Activation System | BISBAS |
| Breastfeeding Self-Efficacy Scale: Short Form with Additional Questions for Critically Ill Infants | BSES-SF-NICU |
| Cognitive and Affective Mindfulness Scale – Revised | CAMS-R |
| Cognitive Emotion Regulation Questionnaire | CERQ |
| Center for Epidemiological Studies–Depression | CES-D |
| Clinical Risk Index for Babies–II | CRIB–II |
| Coping Inventory for Stressful Situations | CISS |
| Davidson Trauma Scale | DTS |
| Depression Anxiety Stress Scales – Short Form | DASS-21 |
| Digi Family-Centered Care: Parent Version | DigiFCC-P |
| Edinburgh Postnatal Depression Scale | EPDS |
| EMpowerment of PArents in THe Intensive Care–Neonatology | EMPATHIC-N |
| EuroQoL 5-dimension 5-level | EQ5D-5L |
| Family-Centered Care Questionnaire | FCCQ |
| Feasibility of Intervention Measure | FIM |
| Five Facet Mindfulness Questionnaire | FFMQ |
| Generalized Anxiety Disorder Scale-7 | GAD-7 |
| Hospital Anxiety and Depression Scale | HADS |
| Impact of Event Scale-Revised | IES-R |
| Infant Care Questionnaire | ICQ or ICC |
| Intolerance of Uncertainty Scale | IUS |
| Inventory of Depression & Anxiety Symptoms–  General Depression Scale | IDAS–GD |
| Inventory of Situations and Responses of Anxiety | ISRA |
| Maslach Burnout Inventory | MBI |
| Maternal Attachment Inventory | MAI |
| Maternal Breastfeeding Evaluation Scale | MBFES |
| Maternal Confidence Questionnaire | MCQ |
| Maternal Postnatal Attachment Scale | MPAS |
| Mini-International Neuropsychiatric Interview | MINI |
| Mother-to-Infant Bonding Scale | MIBS |
| Nurse Parent Support Tool | NPST |
| Parental Stress Scale: Prematurely Born Child | PSS:PBC |
| Parental Stressor Scale | PSS |
| Parental Stressor Scale: Infant Hospitalization | PSS:IH |
| Parental Stressor Scale: NICU | PSS:NICU |
| Parenting Sense of Competence Scale | PSOC |
| Parenting Stress Index | PSI |
| Parenting Stress Index-Short Form | PSI-SF |
| Paternal Postnatal Attachment Scale | PPAS |
| Patient Health Questionnaire 4 | PHQ-4 |
| Patient Health Questionnaire 8 | PHQ-8 |
| Patient Health Questionnaire-9 | PHQ-9 |
| Patient-Reported Outcomes Measurement Information System Adult Profile—Short Form Anxiety | PROMIS Anxiety |
| Perceived Maternal Parenting Self-Efficacy tool | PMP S-E |
| Perceived Stress Scale | PSS-10 |
| Perinatal Posttraumatic Stress Disorder Questionnaire | PPQ |
| Perinatal Risk Inventory | PERI |
| Pittsburg Sleep Quality Index | PSQI |
| Postpartum Bonding Questionnaire | PBQ |
| Postpartum Depression Screening Scale | PDSS |
| Posttraumatic Stress Disorder Checklist for DSM-5 | PCL-5 |
| Quality of Life in Neurological Disorders-Anxiety Short Form | Neuro-QoL-Anxiety Short Form |
| Short Form Health Survey | SF-36 |
| Stanford Acute Stress Reaction Questionnaire | SASRQ |
| State-Trait Anxiety Inventory | STAI |
| State-Trait Anxiety Inventory Short Form | STAI-SF |
| Swedish Parenting Stress Questionnaire | SPSQ |
| Toronto Mindfulness Scale | TMS |
| Traumatic Event Scale | TES; Criterion A |
| Treatment Acceptability and Preference | TAP |
| Yale Inventory of Parental Thoughts and Actions | YIPTA |
