# Supplementary material for: Interventions to support postpartum health and well-being of parents with infants in neonatal intensive care units: a scoping review
Source: Front Health Serv. 2026 Jul 15;6:1845396. doi: 10.3389/frhs.2026.1845396 (PMC13416434; doi:10.3389/frhs.2026.1845396)
Supplement: Supplementary file 3 [file Table3.docx]

| Postpartum NICU Intervention Studies | | | | | |
| --- | --- | --- | --- | --- | --- |
| Author | **Title** | **Country**  **Year Published** | **Intervention** | **Participants** | **Measurement Tool** |
| Mental Health Care | | | | | |
| Kamradt, J.M., Scheiber, F.A., et al | Description of an initiative to optimize mental healthcare services in a level 4 neonatal intensive care unit | United States 2024 | A pre-doctoral psychology trainee and a postdoctoral fellow in psychology with training in perinatal mental health for NICU parents were embedded in the NICU. Supervised by a licensed perinatal psychologist they provided 12 hours per week of on-site care to parents at the baby's bedside or in a private office on the unit. NICU providers referred mothers. Telehealth services were an option. | 79 mothers referred for services. 39 mothers received at least one session. | PHQ-9. GAD-7. Mental health diagnosis Satisfaction survey |
| Shaw, R.J., Moreyra, A., et al | Group Trauma Focused Cognitive Behavior Therapy for Parents of Premature Infants Compared to Individual Therapy  Intervention | United States  2023 | Trauma-focused cognitive behavior therapy delivered over 6 sessions over 3-6 weeks. Sessions offered in a private room in the hospital close to the NICU or by telehealth by a postdoctoral psychology fellow. Results were compared with a previously published study of individual therapy. | 26 mothers of preterm infants who screened positive for depression/ anxiety | PHQ-9. Neuro-QoL-Anxiety Short Form, PPQ, BAI, BDI-II, SASRQ. DTS |
| Segre, L.S., McCabe, J.E. et al | A Randomized Controlled Trial of Listening Visits for Mothers of Hospitalized Newborns | United States 2023 | Nurses who were not the baby's primary nurse provided 60-minute listening sessions during nonwork hours. The mother determined the content of each session. Nurses received training on perinatal depression and reflective listening and problem solving. Sessions were held every 2-3 days in a private hospital location. The control group received treatment provided by the social work team. | 42 mothers with babies in the NICU with mild to moderate distress. | IDAS-GD, CRIB-II, Number of LVs |
| Penny, K.A., Friedman, S.H. et al | Psychiatric support for mothers in the Neonatal Intensive Care Unit | New Zealand 2015 | The Clinical Liaison Team (psychiatrists, psychotherapists, pediatric trainees, social workers, psychologists and nurse specialists) provided psychological and emotional support to referred families. Clinical interviews are unstructured and done at the infant's bedside. | 302 mothers were referred for services | Process measures related to the implementation of the intervention |
| Choi, H.S., Lee, K.E. et al | Effects of an emotional regulation program on maladaptive cognitive emotion regulation, stress, anxiety, and postpartum depression among South Korean mothers of premature infants in the neonatal intensive care unit | South Korea 2021 | Half of the mothers referred had a psychiatric diagnosis. The psychiatrist provided therapeutic work in 56% of the cases. The program created access for a quarter of the mothers to follow-up services. | 38 mothers participated in the study | CERQ, PSS: NICU, STAI, EPDS- Korean Version |
| Shaw, R., St John, N., et al. | Prevention of Traumatic Stress in Mothers of Preterms: 6-Month Outcomes | United States 2014 | The intervention lasted 3-4 weeks with one or two 45–55-minute sessions provided weekly. Sessions incorporated trauma-focused cognitive behavior therapy and infant redefinition. Participants were randomized to control, 6-sessions or 9-sessions. | 105 women with mild / moderate depression with 32 in the 6-session intervention, 25 in the 9-session intervention and 41 in the education session. | Traumatic Events Questionnaire, SASRQ, DTS, BDI-II, BAI, MINI. |
| Fotiou, C., Vlastarakos, P.V. et al | Parental stress management using relaxation techniques in a neonatal intensive care unit: A randomised controlled trial | Greece 2016 | The intervention consisted of five, 90-minute educational sessions where the control group received information on prematurity, stress in the NICU, breastfeeding, and prep for discharge. The intervention group also received information on positive thinking, healthy lifestyle and self-knowledge and practiced 3 relaxation techniques. | 59 parents with 31 in the intervention group and 28 in the control group. There were equal numbers of men and women. | Perceived Stress Scale, STAI, salivary cortisol levels for parents |
| Brady, S., Steinwurtzel, R., et al | Improving Postpartum Depression Screening in the NICU: Partnering with Students to Improve Outreach | United States 2023 | Mothers with babies in the NICU were screened for depression at 2 weeks, 1, 2 and 4 months postpartum. The screening was completed by the bedside nurse. Scores of 10 or more were considered positive and those mothers received support from the NICU care team in making an appointment with a mental health provider and received information and referral resources. | 18 qualifying parents were contacted weekly. Some families received more than one screening. Parents were screened if their infant was 14 or fewer days old. | Percentage of parents who completed the PHQ-9 |
| Brownlee, M.H. | Screening for Postpartum Depression in a Neonatal Intensive Care Unit | United States 2021 | Mothers were screened for depression at 2 weeks, 1, 2 and 4 months postpartum. The screening was completed by the bedside nurse. Mothers with scores of 10 + received support from the NICU care team in making an appointment with a mental health provider and received info and referral resources. | 104 mothers were screened and 53 were not screened. | EPDS, completion of screening, documentation of screening, results and appropriate referral |
| Berns, H.M., Drake, D. | Postpartum Depression Screening for Mothers of Babies in the Neonatal Intensive Care Unit | United States 2021 | Mothers with babies in the NICU for 2 weeks or more were given paper copies of the EPDS. If a mother scored >10 the nurse placed a consult in the EHR for a licensed professional clinical counselor who saw the mother in their private NICU room within 3 days. This project took place over 8 weeks. | 32 mothers were eligible and 25 were screened (78%). | EPDS, process measures around screening and referral |
| Cherry, A.S., Blucker, R.T., et al | Postpartum depression screening in the Neonatal Intensive Care Unit: program development, implementation, and lessons learned | United States 2016 | Mothers with 2-week-old infants hospitalized in a NICU were screened by NICU nursing staff using a paper tool with an envelope and instructions left by the infant's bedside. The project coordinator followed up with mothers to complete the rescreen as well as to review self-harm items and make mental health referrals. | 385 mothers 2+ weeks postpartum were screened | PDSS, process measures around screening and referral |
| Grunberg, V.A., Geller, P.A. | Parental mental health screening in the NICU: a psychosocial team initiative | United States 2022 | Eligible parents were asked by a doctoral-level psychology graduate student at their baby's bedside to complete printed depression screening instruments. The results were reviewed immediately and parents who screened positive were provided with referrals and clinical interventions. The positive screen was flagged for the psychologist and/or social worker for follow up. | 380 families were eligible for mental health screening - 378 mothers and 325 secondary caregivers, including 308 fathers. The parents had infants in the NICU for 5-14 days | CES-D, IES-R |
| Vaughn, A.T., Hooper, G.L. | Development and Implementation of a Postpartum Depression Screening Program in the NICU | United States 2020 | Mothers completed EPDS screening. Assessments were scored within an hour. Diagnostic referral reference handouts were provided to any mother who scored 10 or higher - they also received a follow up within two weeks. | 30 mothers with hospitalized infants > 30 days of age | EPDS, process measures around screening and referral |
| Moreyra, A., Dowtin, L.L. et al | Implementing a standardized screening protocol for parental depression, anxiety, and PTSD symptoms in the Neonatal Intensive Care Unit | United States 2021 | Parents received a 30-minute consultation to complete screening and introduce mental health services in the NICU. Screening was done by paper or electronic tablet. When a parent had a positive screen a NICU psychology fellow and attending psychologist or psychiatrist conducted a one-hour semi-structured clinical interview. Results were documented in the infants' EHR unless the parents described a confidential history or suicidal ideation in which case a separate chart was created. | 150 parents with an infant in the NICU at least two weeks completed screens | PHQ-9, Neuro-QoL-Anxiety Short Form, PPQ, process measures |
| Grieb, S.M., McAtee, H. et al | Exploring the Influence of a Mindfulness Intervention on the Experiences of Mothers with Infants in Neonatal Intensive Care Units | United States 2023 | Mothers received one set of mindfulness related material early in the infant's NICU stay. The materials included a 20-minute introductory video and audio recordings of 4 mindfulness topics between 5-10 minutes long. The second set of materials was provided before the infant's discharge and consisted of an intro video and four short mindfulness videos. Participants were contacted 1-2 days after they viewed the initial video with a second check in a week later regarding questions. | 26 mothers whose infants were expected to be hospitalized for at least 2 weeks Mothers with serious mental health concerns were excluded. | two semi-structured in-depth interviews per participant |
| Russell, L.N., Gregory, M.L. et al | Uptake and impact of journaling program on wellbeing of NICU parents | United States 2021 | Parents in the intervention group received a journal with instructions to use the journal as frequently as they chose. Journal content was not reviewed by study staff. Parents could choose to share topics they wrote about but the entries were not read or evaluated. The journals had blank pages for free text as well as prompts to guide parents if needed. The intervention was 2-4 weeks. | 36 mothers and 14 fathers were in the intervention group with infants hospitalized in the NICU with at least 5 days of anticipated stay. | HADS, self-report on social media use, mental health treatment, education, # of other children at home and prior NICU experience, process measures on use of journals, parent experience with the journal |
| Marshall, A., Guillen, U., et al. | Mindfulness Training among Parents with Preterm Neonates in the Neonatal Intensive Care Unit: A Pilot Study | United States 2019 | Parents who consented to the intervention received a mindfulness-based training session in a private room (not at the baby's bedside). The sessions were ~1 hour and provided parents with formal instruction and practice on several mindfulness techniques. Parents were given links to free downloadable mindfulness programs. They had access to study staff for questions and access to mindfulness meditations on an iPad kept in the NICU. | 36 parents completed sessions and 28 also completed the surveys. | PSS: NICU, CAMS-R, survey on stress management techniques |
| Petteys, A.R., Adoumie, D. | Mindfulness-Based Neurodevelopmental  Care Impact on NICU Parent Stress and Infant Length of Stay; A Randomized  Controlled Pilot Study | United States 2018 | Parents in the intervention group received a one-on-one educational session where they were taught mindfulness techniques and neurodevelopmental care training centered on observing and responding to infant cues. The session lasted from 30-60 minutes. Parents then had contact with a research team member at least every other week for the duration of their baby's NICU stay where they could ask questions. | 55 parent-infant dyads | Infant clinical information, general demographic form, PSS: NICU, MIBS, Parent Satisfaction Score, Parent-Infant Interaction Log |
| Mendelson, T., McAfee, C., et al | A mindfulness intervention to reduce maternal distress in neonatal intensive care: a mixed methods pilot study | United States 2018 | While there were no statistically significant differences between the groups, parents in the experimental group had a reduction in stress scores and their infants had a shorter length of stay. | 24 women with infants with an anticipated hospital stay of at least 2 weeks but not experiencing an acute medical crisis or at risk of death. | PHQ-8, GAD-7, SASRQ, PSQI, PSS: NICU, Brief COPE, FFMQ, MIBS, Self-Compassion Scale, post-intervention interviews |
| Horsch, A., Gilbert, J., et al | Improving Maternal Mental Health Following Preterm Birth Using an Expressive Writing Intervention: A Randomized Controlled Trial | Switzerland 2016 | Mothers were recruited while their baby was in the NICU and contacted when their baby was 3 months corrected age. The intervention group received a workbook with instructions and prompts, encouraging them to process their thoughts and feelings linked to their baby's NICU stay. Participants were asked to write for 15 minutes over 3 consecutive days. | 67 mothers were recruited. 54 mothers completed the 6-month surveys. | PPQ, EPDS, SF-36, Use of healthcare services, satisfaction with the intervention, CRIB-II, PERI |
| Kobus, S., Diezel, M., et al | Music Therapy in Preterm Infants Reduces Maternal Distress | Germany 2022 | Mothers of preterm infants born before 32 weeks’ gestation were enrolled in a NICU-based randomized trial comparing infant-directed music therapy plus standard care to stand care alone. From 2 weeks until discharge, infants in the intervention arm received two music therapy sessions per week. Mothers were assessed with the CES-D and IES-R one week after birth and at discharge. | 33 mothers with 18 mothers in the music therapy group, and 15 in the control group. | Center for Epi Studies Depression Scale (German), Post-traumatic stress symptoms - IES-R |
| Meke, B., Hass, J., et al | Family-centered music therapy—Empowering  premature infants and their primary caregivers through music: Results of a pilot  study | Germany 2021 | The study recruited parents and infants born less than 30 weeks and very preterm term. Treatment group received music therapy twice weekly from day 21 of life until discharge and the control group received standard care. | 65 Parent-infant pairs. 47 treatment (26 mothers, 21 fathers), 43 in control (18 mothers, 17 fathers) | PSS:PIC, STAI, EPDS, and MIBS |
| Kraft, K., Jaschke, A., et al | Maternal Anxiety, Infant Stress, and the Role of Live-Performed Music Therapy during NICU Stay in The Netherlands | Netherlands  2021 | Mothers of infants born before 30 weeks were included in a prospective cohort study during NICU stays, with a randomized-controlled trial of live performed music therapy (LPMT) versus waitlist control. Infants received LPMT sessions in the weeks after birth, while maternal anxiety was tracked longitudinally, showing a natural decrease over time accelerated by the intervention (12% reduction post LPMT versus 1% increase in controls). | 45 mother-infant dyads; 21 mothers completed STAI before and after the LPMT (n=12) or waitlist (n=9) | STAI, NISS |
| Postpartum Physical and Social Care | | | | | |
| Cordova-Ramons, E.G., Jain C. et al | Implementing Social Risk Screening and Referral to Resources in the NICU | United States 2023 | Integrate screening and referral for social determinants of health into NICU clinical workflow. The screening tool was adapted from an existing tool, "THRIVE" which was integrated into the health care system's EHR. The resource guide was co-created by social workers and family navigators following guidance from the American Academy of Pediatrics. | 103 Families | 49% of families screened for 7 social risk factors within 2 weeks of admission. 52% unmet needs for which families connected with resources within 3 months of referral. 98% of families with one or more unmet needs that were provided with a resource guide. 0% families that declined screening |
| Massa, K., Ramireddy, S. et al | A Randomized Control Trial of Meditation for Mothers Pumping Breastmilk for Preterm Infants | United States 2022 | Mindfulness-based meditation can be an effective way to reduce stress, improve mood and support new behaviors. Women in the intervention received access to the Expectful app and asked to use the app, designed for pregnancy, postpartum and motherhood, daily while expressing breast milk. | 60 mothers who were providing breastmilk to their babies | Mean volume of expressed milk in 24 hours. EPDS; STAI, PSS-NICU, BSES-SF-NICU. Breastmilk provision at 28th day of life. Lactating promoting behaviors - skin to skin contact, frequent pumping and hand expression with pumping by self-report. |
| Ericson, J., Eriksson, M., et al | Proactive telephone support provided to breastfeeding mothers of preterm infants after discharge: a randomised controlled trial | Sweden 2018 | Proactive daily telephone call provided to mothers who were breastfeeding preterm infants from day one until day 14 after NICU discharge. Mothers were encouraged to discuss whatever they felt important. All mothers in the study had the option to call for support (reactive telephone support). | 493 women who were breastfeeding | SF-36. Exclusive breastfeeding. MBFES. MPAS. SPSQ |
| Verbiest, S., McClain, E. et al | Postpartum Health Services Requested by Mothers with Newborns Receiving Intensive Care | United States 2016 | A certified nurse midwife provided health care services to NICU mothers during week day work hours. | 424 health care encounters for women with NICU babies | Health Care Encounters - service, duration, location of care |
| Stotts, A.L., Northrup, T.F. et al | Reducing Tobacco Smoke Exposure in High-Risk Infants: A Randomized, Controlled Trial | United States 2020 | This study compared motivational interviewing (two, 30-minute sessions in the hospital by masters prepared counselors with two additional sessions 2 weeks a part in the participants' homes starting 2 weeks after NICU discharge) paired with financial incentives with regular care for mothers of infants in the NICU who reported a smoker living in the home. | 360 primary care givers (mothers) | Infant urine cotinine level. Adapted contemplation ladder, self-reported home and car smoking bans, air-nicotine levels, Timeline FollowBack, CO2 breath sample, mother reported partner smoking, attendances at counseling sessions, infant healthcare use. |
| Northrup, T.F., Suchting, R., et al | Proactive delivery of nicotine replacement therapy to families of hospitalized infants in a NICU: A randomized controlled pilot trial | United States 2020 | This pilot study considered the feasibility and efficacy of providing motivational advice (2 in-hospital sessions) and nicotine replacement therapy (NRT) (2-weeks of either 14-mg or 21-mg trasndermal patches for every smoker in the house) to families of NICU infants compared to Quitline referral. Partners and others in home who smoked were invited to attend. | 32 participants, most were mothers and nonsmokers (84.4%). Other household members (n=7) attended session 1 and session 2 visits (n=3) | Baseline assessment visit (in the hospital) and one FU assessment visit at the hospital or by phone at 2 weeks and 1 month post intervention. Sociodemographic and smoking related questions and a Timeline-Followback Interview modified to assess NRT usage and smoking by all household smokers. Contemplation Ladder. Plans to quit in the next 30 days, 24-hour quit attempts, readiness to use NRT, smoking bans, accepting/use of NRT. |
| Dunlop, A., Logue, K. et al | Maternal Health Risk Assessment and Behavioral Intervention in the NICU Setting Following Very Low Birth Weight Delivery | United States 2016 | Women who had given birth to a very low birthweight baby received either a minimal intervention with a single session risk assessment and health education pamphlets or an enhanced intervention and received 5 counseling sessions focusing on identified risks. | 80 mothers of VLBW infants | Medical chart abstraction. Your Reproductive Plans and Health Risks questionnaires Timeline Follow Back for folic acid use and unprotected intercourse, attitudinal survey about experiences with intervention. Baseline, 3 and 9 months post intervention |
| NICU Design and Technology | | | | | |
| Campbell-Yeo, M; Kim, T; et al. | Do Single-Family Rooms Increase Parental Presence, Involvement, and Maternal Well-Being in Neonatal Intensive Care? | Canada 2021 | The NICU was under renovation to transition from open-bay (OB) setting to single-family rooms (SFR). Participants were assigned to either the OB or SFR spaces. | 71 mothers | Daily diary sheet, PDSS, PSS: NICU, EQ5D-5L, PCL-5, PMP S-E, IUS |
| Andersen, K; Holm, K; et al. | Association between neonatal homecare for preterm infants and incidence of severe postpartum depression in mothers | Denmark 2021 | Neonatal homecare was introduced in 17/19 Danish NICUs from 1997-2016. This study used an interrupted time series analysis to measure the effect of neonatal homecare on severe postpartum depression in mothers. The team used national register data for this analysis. | 46456 mothers | Diagnosis with ICD-10 code at a psychiatric hospital (inpatient or outpatient) or obtained a prescription for antidepressant medication within the first six months postpartum |
| Neri, E; Genova, F; et al. | Parental Distress and Affective Perception of Hospital Environment after a Pictorial Intervention in a Neonatal Intensive Care Unit | Italy 2022 | Parents’ experiences of distress and their perception of the NICU environment were considered based on exposure to a pictorial intervention in the form of a colorful mural in part of the NICU space. | 48 parents (38 mothers and 10 fathers) | Rapid Stress Assessment Scale (VRS); QAL |
| Kirolos, S; Sutcliffe, L; et al. | Asynchronous video messaging promotes family involvement and mitigates separation in neonatal care | United Kingdom 2020 | Evaluation of both the parent and staff experience of using the vCreate Neonatal Video Diary service. This asynchronous video service allowed for parents to receive general updates and key moments that they were not able to be present for. | Post-implementation surveys - 42 families, 77 staff | Pre- and post-implementation surveys for staff and parents. |
| Legge, A; Middleton, J; et al | Implementation of a Web Camera System in an Australian Neonatal Intensive Care Unit: Pre- and Postevaluation of the Parent and Staff Experience | Australia 2023 | This intervention measured the attitudes of parents and staff before and after a web-based camera system was implemented in the NICU as well as changes in parent stress and depression. | 125 parents and 110 staff completed pre-surveys - 94 parents and 109 staff completed post-surveys | Pre- and post-implementation surveys for staff and parents. DASS-21; PSS: NICU (only in post). |
| Ahn, H; Jo, H. | Effects of a Noncontact Visit Program in the NICU for the Prevention of COVID-19 | South Korea 2023 | A non-contact visit program for NICU parents was developed to prevent COVID-19 infection and address nursing and parental stress | 36 mothers | Nurse–Parent Support Tool, PSS; PSS: NICU |
| Reimer, A; Mause, L; et al | Webcam use in German neonatological intensive care units: an interview study on parental expectations and experiences | Germany 2021 | Qualitative study of parents' perspectives about the implementation of webcams in the NICUs | 33 mothers, 7 fathers | Parent interviews |
| Kubicka, Z; Zahr, E; et al | Use of an internet camera system in the neonatal intensive care unit: parental and nursing perspectives and its effects on stress | United States  2021 | The study evaluated parent and nurse perspectives of stress when webcams were and were not available in the NICU | 79 parents off webcam, 80 parents on webcam, 35 nurses | Parent questionnaire: PSS NICU |
| Kerr, S; King, C; et al | Transition to parenthood in the neonatal care unit: a qualitative study and conceptual model designed to illuminate parent and professional views of the impact of webcam technology | Scotland 2017 | The study evaluated parent and professional perspectives of the mylittleone webcam intervention. This intervention allowed parents to see video footage (not including procedures) of the baby in the unit before the mother was discharged from the postnatal setting. | 33 parents - 25 mothers and 8 fathers; 18 professionals | Parent and professional interviews |
| Tandberg, B; Flacking, R; et al | Parent psychological wellbeing in a single-family room versus an open bay neonatal intensive care unit | Norway 2019 | The study compared the experiences of parents in single family rooms (SFR) and parents in an open bay (OB) NICU. The SFR experience was in a different hospital, and the setting provided two beds for parents that could be behind a partition, a private bathroom, and free meals provided to parents. The SFR unit had a psychologist available to parents part-time and weekly meetings with other NICU parents. In the OB unit, parents could not stay overnight, there was one armchair for parents, mothers could stay in a different building at the hospital after discharge if requested, free meals were available to mothers, and a psychologist was available if requested. | 132 parents (72 OB, 60 SFR) | time spent in NICU, EPDS, STAI SF, PSS: NICU, PSI-SF, MPAS |
| Infant Care and Development Education | | | | | |
| Chen, Y; Lee, T; et al | The Effectiveness of an Intervention Program for Fathers of Hospitalized Preterm Infants on Paternal Support and Attachment 1 Month After Discharge | Taiwan  2019 | Fathers in the intervention group received additional educational materials about caring for their infant and were able to engage with the nurse for teaching, receive support and guidance, encourage activities and to ask questions. | 82 fathers | PSS: IH, fathering ability scale, paternal support scale, MAI |
| Cano Gimenez, E; Sanchez-Luna, M | Providing parents with individualised support in a neonatal intensive care unit reduced stress, anxiety and depression | Spain  2015 | A 5 step intervention in which parents in the NICU engaged in activities including orientation to the NICU setting and procedures, guidance, education, coping support, and planning support | 80 mothers, 54 fathers | PSS: NICU, EPDS, ISRA, BDI |
| Borghini, A; Habersaat, S; et al | Effects of an early intervention on maternal post-traumatic stress symptoms and the quality of mother-infant interaction: The case of preterm birth | Switzerland  2014 | The study assessed how a three part intervention program that included join infant observation by the parent, nurse and therapist, assessment of the Neonatal Behavioural Assessment Scale (NBAS) and an interview with the mother using e Clinical Interview for Parents of High-Risk Infants, and videotaped interactions between the mother and infant followed by guidance affected maternal posttraumatic stress and quality of maternal infant interactions | 78 mothers and their infants | PPQ, CARE-Index |
| Hoffenkamp, H; Tooten, A; et al | Effectiveness of Hospital-Based Video Interaction Guidance on Parental Interactive Behavior, Bonding, and Stress After Preterm Birth: A Randomized Controlled Trial | Netherlands  2015 | Parents engaged in Video Interaction Guidance and received feedback about their interactions with infants. The study evaluated the effect of VIG on bonding, sensitivity, mental health, and trauma. | 150 families (150 infants, 150 mothers, 144 fathers) | Coding of video interactions in categories of Parental Sensitivity, Parental Intrusiveness, Parental Withdrawal, PBQ, My Baby and I questionnaire (MBI), YIPTA, PSS: NICU, EPDS, TES; Criterion A |
| Fratantoni, K; Soghier, L; et al | Giving parents support: a randomized trial of peer support for parents after NICU discharge | United States  2022 | Parents in the control group received a care notebook with information to support them in caring for their infant after discharge. Parents in the intervention group also received the care notebook, in addition to a peer navigator that supported them in identifying resources, communicating with providers, providing emotional support, and more in the year after discharge. | 150 allocated to intervention and 150 to control (participant included infant and primary caregiver) | PSS-10, Parental Stress Scale (PSS), PMPS-E, CES-D 10, STAI Y-1, Infant outcomes (ED visits/hospitalizations, immunizations, infant developmental outcomes (Bayley Scales of Infant and Toddler Development®, Third Edition) |
| Ji, E; Shim, K | Effects of a Community-based Follow-up Program for Parents with Premature Infants on Parenting Stress, Parenting Efficacy, and Coping | South Korea  2020 | Participants in the control group received standard home visiting by community visiting nurses. Participants in the experimental group received home visiting by an experienced NICU nurse and a community visiting nurse with more elements included in the physical exam than in standard home visiting (other elements were the same). Additionally, participants in the experimental and control groups engaged in support group meetings. | 29 in experimental group and 27 in control group included in analysis | Parenting Stress Index (PSI), Parenting Sense of Competence (PSOC), Coping Health Inventory for Parents (CHIP) |
| Gibson, C; Williams, M; et al | Distress, self-efficacy, feeling informed and the Babble app: A New Zealand neonatal parent sample | New Zealand  2023 | The study assessed whether the Babble App impacts parents feelings of self-efficacy, distress, and feeling informed. | 449 parents (438 mothers), 44 participants used the Babble App | PSS: NICU, novel single self-efficacy question measured on a 5-point Likert scale, novel set of 4 questions with Likert scale response to assess how informed parents felt |
| Collette, K; Feeley, N; et al | Acceptability and feasibility of a digital educational intervention designed to improve the psychological well-being of parents with a preterm infant at the neonatal intensive care unit: A pilot project | Canada  2023 | This pilot RCT assessed the feasibility of the study design and data collection, as well as participant reaction to a French language website about the NICU in the intervention group compared to an electronic pamphlet about the NICU, and how the website affected the parent psychological wellbeing. | 13 participants in intervention group, 7 in control group (19 mothers 1 father) | TAP Questionnaire plus additional questions added, PSS: NICU French version,  EPDS French version |
| Erdei, C; Forde, M; et al | “My Brigham Baby” Application: A Pilot Study Using Technology to Enhance Parent's Experience in the Neonatal Intensive Care Unit | United States  2024 | This pilot study assessed the feasibility of an educational app (the My Brigham Baby app) for support in the NICU setting and the impact of the My Brigham Baby app on parental psychosocial experience while their infant was in the NICU. The app contains resources including information about the NICU experience and external supports, communication with the care team, and some medical information about the infant. | 50 parents (25 pre-App and 25 post-App rollout) | Survey included discharge readiness, PSOC, PSS: NICU, GAD-7 |
| Dahan, S., Bourque, C.J., et al | Community, Hope, and Resilience: Parental Perspectives on Peer Support in Neonatology | Canada  2022 | Ten consecutive weekly peer-to-peer meetings moderated by two resource parents and parents of infants hospitalized in the NICU. The meetings were one hour with a stable format, include a list of potential themes for discussion. NICU providers were not present. | 45 NICU parents attended the meetings - 14 fathers, 31 mothers and 3 other family members. 39 parents completed the questionnaire. | Post-meeting questionnaires asking parents to rate the meeting and respond to open ended questions. |
| Family-Centered Care | | | | | |
| Axelin A, Feely N, et al | Symptoms of depression in parents after discharge from NICU associated with family-centred care | Australia, Belgium, Canada, Croatia, Denmark, Estonia, Finland, Iceland, Lithuania, the Netherlands, Norway, Poland, Spain, Sweden,  United Kingdom  2022 | The intervention was the delivery of Family Centered Care by the NICU. Parental perception of FCC was assessed daily while in the NICU by the DigiFCC-P via text and the FCCQ:Parent Version near discharge. Depression was assessed via EPDS at discharge and 4 months infants' corrected age. | 635 mothers and 466 fathers of 739 infants born before 35 weeks gestation and admitted to a participating NICU | EPDS; DigiFCC-P; FCCQ: Parent version |
| DeBernardo G, Svelto M, et al | Supporting parents in taking care of their infants admitted to a neonatal intensive care unit: a prospective cohort pilot study | Italy  2017 | Infants admitted to NICU and their parents were divided into two cohorts: 8 hours/day parental access to infants and FCC approach and 1 hour/day parental access to infants and standard approach to care | 48 mothers and 48 fathers of 48 singletons from Naples | PSS: NICU; Parental satisfaction survey validated by Abdel-Latif ME et al. |
| DeBernardo G, Napolitano G, et al | Improving Care in Neonatal Intensive Units During the COVID-19 Pandemic: A Survey on Electronic Health Communication | Italy  2022 | Intervention was a version of the FCC approach that involved video and telephonic calls and no access to NICU (TFCC), as compared to 8 hours of in-person access to NICU infant +FCC approach (FCC), and to 1 hours access to infant and non-FCC approach (NFCC). | 136 parents of NICU infants (68 mothers, 68 fathers) | PSS-HI; Parental satisfaction survey validated by Abdel-Latif ME et al. |
| Franke L, Gay C, et al | Maternal mental health after infant discharge: a quasi-experimental clinical trial of family integrated care versus family-centered care for preterm infants in U.S. NICUs | United States  2023 | Infants <33 weeks gestation & their parents in 3 participating NICUs were divided into two groups that received Family-Centered Care (FCC) or mobile-enhanced Family-Integrated Care (mFICare), and the effect on post-discharge maternal mental health symptoms was assessed. | 178 mothers | PPQ; EPDS; PSS: NICU; DigiFCC-P |
| Ahlqvist-Bjorkroth S, Axelin A, et al | An educational intervention for NICU staff decreased maternal postpartum depression | Finland  2019 | The intervention was the delivery of the Close Collaboration with Parents program which educates all of the NICU staff on collaborating with parents, and the effect on maternal depression at 4-6 months postpartum was assessed. | 238 mothers of preterm infants < 1500g, with no major congenital anomalies or syndromes, discharged alive | EPDS |
| Horner S, Benbrook K, et al | Implementing Guidelines for NICU Parent Presence: Effects on Parent and Infant Stress | United States  2023 | Intervention was implementing the communication of specific guidelines around parental presence in the NICU, asking at least 1 parent to be present at least 4 hours/day | 78 NICU families (40 infants, their mothers, and 20 fathers preintervention compared to 38 infants, their mothers, and 15 fathers post-intervention) | PSS: NICU |
| McLean MA, Scoten OC, et al | Lower Maternal Chronic Physiological Stress and Better Child Behavior at 18 Months: Follow-Up of a Cluster Randomized Trial of Neonatal Intensive | Canada  2022 | Family Integrated Care (FICare) vs standard of care, randomized by NICU | 126 mother-child dyads (FiCare sites: n=83; standard of care sites: n=43) | PSI Long Form (4th ed), Parent Domain; STAI; hair cumulative cortisol [HCC] and dehydroepiandrosterone [DHEA) from hair samples |
| Cheng C, Franck LS, et al | Evaluating the effect of Family Integrated Care on maternal stress and anxiety in neonatal intensive care units | Australia, Canada, New Zealand  2021 | Family Integrated Care (FICare) vs standard of care, randomized by NICU | 1,383 mothers (control: n= 673; FiCare: n=710) | PSS: NICU; STAI |
| O'Brien K, Robson K, et al | Effectiveness of Family Integrated Care in neonatal intensive care units on infant and parent outcomes: a multicentre, multinational, cluster-randomised controlled trial | Australia, Canada, New Zealand  2018 | Family Integrated Care (FICare) vs standard of care, randomized by NICU | Parents of 1,786 infants (FiCare sites: n=895 infants; Standard of care sites: n=891) | PSS: NICU; STAI |
| van Veenendaal NR, van Kempen AAMW, et al | Association of a Zero-Separation Neonatal Care Model With Stress in Mothers of Preterm Infants | Netherlands  2022 | Family Integrated Care (FICare) with couplet care for mother-infant dyads in private rooms vs standard of care in open bay NICUs | 296 mothers, of whom 239 answered questionnaires | PSS: NICU; HADS; PMPS-E; PBQ; EMPATHIC-N; CO-PARTNER tool |
| van Veenendaal NR, van der Schoor SRD, et al | Association of a Family Integrated Care Model With Paternal Mental Health Outcomes During Neonatal Hospitalization | Netherlands  2022 | Family Integrated Care (FICare) with couplet care for mother-infant dyads in private rooms vs standard of care in open bay NICUs | 263 fathers, of whom 182 answered questionnaires | PSS-NICU; HADS; PMPS-E; PBQ; EMPATHIC-N; CO-PARTNER tool |
| Stelwagen M, van Kempen A, et al | Parents' Experiences With a Model of Integrated Maternity and Neonatal Care Designed to Empower Parents | Netherlands  2021 | Family Integrated Care (FICare) with couplet care for mother-infant dyads in private rooms | 36 parents (27 mothers, 9 fathers) of infants in NICU at least 7 days | 4 focus groups and 8 semi-structured interviews, 1-3 months post discharge |
| Ansari NS, Franck LS, et al | A Pilot Study of Family-Integrated Care (FICare) in Critically Ill Preterm and Term Infants in the NICU: FICare Plus | Canada  2023 | Family Integrated Care (FICare) Plus: implementation of FICare for critically ill infants of all gestational ages | 41 parents of extremely low birth weight infants on invasive positive-pressure ventilation for >48 h after birth, infants with surgical necrotizing enterocolitis or bowel perforation, infants with tracheoesophageal fistula or esophageal atresia (FICare Plus: n=24 = 20 mothers, 4 fathers; Standard care: n=17 = 14 mothers, 3 fathers) | PSS: NICU; STAI; Perceived Parenting Self-Efficacy Tool; Family Centered Care Survey |
| Kubicka Z, Fiascone J, et al | Implementing modified family integrated care in a U.S. neonatal intensive care unit: nursing perspectives and effects on parents | United States  2023 | Implementation of Family Integrated Care (FICare) without a required number of hours of parental presence at bedside in a NICU - comparison of pre- and post-implementation | 169 parents of infants in the NICU (79 pre-implementation, 90 post-implementation) | PSS: NICU |
| Nieves H, Clements-Hickman A, et al | Effect of a Parent Empowerment Program on Parental Stress, Satisfaction, and Length of Stay in the Neonatal Intensive Care Unit | United States  2021 | Nurse-implemented "Creating Opportunities for Parent Empowerment” (COPE) program | 49 mothers of NICU infants <35 weeks gestation (Control: n=29; Intervention: n=20) | PSS: NICU; EPDS; investigator-designed Baptist Health Lexington parent satisfaction survey |
| Ahlqvist-Björkroth S, Axelin A, et al | Fewer maternal depression symptoms after the Close Collaboration with Parents intervention: Two-year follow-up | Finland  2022 | Implementation of the Close Collaboration with Parents NICU staff education program | 180 mothers of VLBW (<1501g) infants without major congenital anomalies who were alive at discharge (Pre-implementation: n=126; Post-implementation: n=54) | EPDS |
| LeDuff III LD, Carter BM, et al | NICU Fathers: Improving the Quality of Paternal Support in the NICU | United States  2020 | Nursing educational program designed to improve NICU fathers' perception of nursing support provided to them. | 52 fathers of infants in the NICU | NPST |
| Parent-Infant Interaction Enhancement | | | | | |
| Freccero A, Scala M, et al | The Safety of Body Wraps on Skin-to-Skin Care in the Neonatal Population | United States  2024 | The use of body wraps during skin-to-skin care (SSC) was compared to standard of care. | 29 parent-infant dyads with infants younger than 34 weeks post-menstrual age and eligible to be held according to the NICU's algorithm | PSS; Parental feedback form |
| Saltzman AM, Sigurdson K, et al | Barriers to Kangaroo Care in the NICU: A Qualitative Study Analyzing Parent Survey Responses | United States  2022 | Qualitative survey to assess parental feelings about kangaroo care (KC) and perceived barriers to engaging in KC | 50 NICU parents with at least 5 days in the NICU and at least one experience of KC with their infant. | Researcher-developed survey |
| Cristóbal-Cañadas D, Parrón-Carreño T, et al | Effect of the Kangaroo Mother Method after Preterm Delivery on Maternal Stress and Anxiety in the Context of the COVID-19 Pandemic: A Cohort Study | Spain  2022 | All mothers in the study agreed to engage in Kangaroo Mother Method (KMM) of at least 90 min for 12 days. | 112 mothers of 28-34 weeks gestation infants in the NICU | PSS: NICU; STAI |
| Zych B, Błaż W, et al | Perception of Stress and Styles of Coping with It in Parents Giving Kangaroo Mother Care to Their Children during Hospitalization in NICU | Poland  2021 | Implementation of Kangaroo Mother Care (KMC) in NICU | 337 parents (mothers = 261; fathers = 76) of preterm infants in NICU | PSS: NICU; CISS |
| Lisanti AJ, Demianczyk AC, et al | Skin-to-Skin Care is Associated with Reduced Stress, Anxiety, and Salivary Cortisol and Improved Attachment for Mothers of Infants With Critical Congenital Heart Disease | United States  2021 | Implementation of skin-to-skin care (SSC) before and after neonatal cardiac surgery | 30 biological mothers of full-term infants with critical congenital heart disease hospitalized within 1 week of birth for neonatal cardiac surgery | PSS: IH; STAI; researcher-designed visual analog scales to assess current stress, current attachment, and current perceptions of infant fragility;  CES-D;  MAI; salivary cortisol; salivary oxytocin |
| Jones H, Santamaria N | Physiological benefits to parents from undertaking skin-to-skin contact with their neonate, in a neonatal intensive special care unit | Australia  2017 | Skin-to-skin care (SSC) | 26 parents (22 mothers, 4 fathers) of NICU infants currently 28-35 weeks gestation during SSC and receiving oxygen therapy on respiratory support | Assessment of parental heart rate and blood pressure 15 and 30 minutes into a SSC session. |
| Sweeney S, Rothstein R, et al | Impact of kangaroo care on parental anxiety level and parenting skills for preterm infants in the neonatal intensive care unit | United States  2017 | Parents engaged in at least 2 kangaroo care (KC) sessions lasting at least 30 minutes | 116 parents of NICU infants born ≤34 weeks gestation and ≤2500g | STAI; 1 question parental readiness assessment about comfort level at discharge |
| Cho ES, Kim SJ, et al | The Effects of Kangaroo Care in the Neonatal Intensive Care Unit on the Physiological Functions of Preterm Infants, Maternal-Infant Attachment, and Maternal Stress | South Korea  2016 | Kangaroo Care (KC) sessions of 30 min, 3 times per week for 10 sessions. | 40 mothers of infants ≥33 weeks, graduated from respiratory support (control=20; intervention = 20) | PSS: NICU adapted by Jeon; MAI adapted by Han |
| Samra HA, Dutcher J, et al | Effect of Skin-to-Skin Holding on Stress in Mothers of Late-Preterm Infants | United States  2015 | Skin-to-skin care (SSC) 50 min per session at least 3 times/week vs blanket-wrapped infant held at least 50 min at least 3 times/week | 30 mothers of late preterm infants (born 34-36 6/7 weeks gestation) in the NICU (19 intervention; 11 control) | PSS: NICU |
| Landry MA, Kumaran K, et al | Mindful Kangaroo Care: mindfulness intervention for mothers during skin-to-skin care: a randomized control pilot study | Canada  2022 | Mothers were taught 2 mindfulness exercises to practice during Kangaroo Care (MKC) through 2 weekly coaching sessions and 2 weekly follow-up sessions vs standard of care including KC (control) | 30 mothers (15 MKC; 15 control) | PSS-NICU; PHQ4, TMS; researcher-designed acceptability assessment |
| Holditch-Davis D, White-Traut RC, et al | Maternally Administered Interventions for Preterm Infants in the NICU: Effects on Maternal Psychological Distress and Mother-Infant Relationship | United States  2014 | Auditory-tactile-visual-vestibular (ATVV) intervention vs kangaroo care (KC) vs attention control intervention where study nurse spent time with mother on how to select & locate equipment to care for infant at home (Attention Control) | 240 mothers of preterm infants born ≤1740g (Attention Control = 81; ATTV = 78; KC = 81) | CES-D; STAI; PPQ; PSS: PBC; The Worry Index; Vulnerable Child Scale |
| Kostilainen K, Mikkola K, et al | Effects of maternal singing during kangaroo care on maternal anxiety, wellbeing, and mother-infant relationship after preterm birth: a mixed methods study | Finland  2021 | Certified music therapist guided mothers to sing or hum during Kangaroo Care (Singing KC) during gestational weeks 33-40 vs KC without encouragement to sing/hum (control) | 36 mothers (intervention = 24; control = 12) of | STAI; researcher-designed questionnaire about intervention |
| Vittner D, McGrath J, et al | Increase in Oxytocin From Skin-to-Skin Contact Enhances Development of Parental-Infant Relationship | United States  2018 | Saliva was collected from parents and infants before, during, and after 60-minute skin-to-skin contact session (SSC). Parents also filled out a Visual Anxiety Scale immediately after each saliva collection. | 28 mother- father-infant triads of infants in the NICU (27 fathers) | Salivary cortisol; Salivary oxytocin; Visual Anxiety Scale |
| Noergaard B, Ammentorp J, et al | Fathers' Stress in a Neonatal Intensive Care Unit | Denmark  2018 | Implementation of a "Father Friendly NICU," which included encouraging fathers to participate in care, scheduling medical updates when both parents can attend, holding father support groups, having social workers counsel fathers about paternity leave and other socioeconomic supports, the inclusion of an additional support adult beyond the parents, and allowing siblings to spend the night | 109 fathers of NICU infants (Control = 55; Intervention = 54) | PSS: NICU |
| McCarty D, Silver R, et al | Infant Massage as a Stress Management Technique for Parents of Hospitalized Extremely Preterm Infants | United States  2024 | Physical therapist taught mothers how to perform infant massage, including modeling on a doll while providing oral instructions, leaving written instructions, and observing mothers provide infant massage at least twice. | 22 mother-infant dyads; infants born ≤ 28 weeks gestation; 33-34 weeks post menstrual age at time of intervention | Salivary cortisol |
| Pineda R, Wallendorf M, et al. | A pilot study demonstrating the impact of the supporting and enhancing NICU sensory experiences (SENSE) program on the mother and infant | United States  2020 | Implementation of the SENSE program, which includes specific doses of parent-administered touch, auditory stimulation, visual/light stimulation, touch, and vestibular and olfactory stimulation. | 73 infants and their mothers (46 standard of care; 27 intervention) who were born <32 weeks and enrolled in the SENSE study in the first week of life | PSS; STAI; EPDS; Modified PPQ; PSS: NICU Parental Role Alterations subscale; MCQ; ICC |
| Richter M, Angell A, et al | Infant and Parent Outcomes Related to NICU-Based Co-occupational Engagement | United States  2023 | Comparison of SENSE intervention administration by parents vs. volunteers/health care providers on infant neurobiological outcomes and parental outcomes. | 35 infants born <32 weeks and enrolled in the SENSE study within the 1st week of life and had engagement in the intervention (28 parent-administered, 7 volunteer/ healthcare administered) | STAI; EPDS; PSS); PSS NICU; PSI; MCQ; ICQ |
| McCarty DB, Dusing SC, et al | A Feasibility Study of a Physical and Occupational Therapy-Led and Parent-Administered Program to Improve Parent Mental Health and Infant Development | United States  2023 | Study of feasibility and acceptability for parents of very preterm infants of Therapist Education and Massage for Parent-Infant Outcomes (TEMPO) program | 25 parents (24 mothers, 1 father) who can speak & understand English of 27 infants born <28 weeks gestation; enrolled < 4 weeks of life | PROMIS Anxiety; CES-D; EPDS; PSOC; MPAS; PPAS; AIM; FIM |
| Cañadas DC, Carreño TP, et al | Benefits of Kangaroo Mother Care on the Physiological Stress Parameters of Preterm Infants and Mothers in Neonatal Intensive Care | Spain  2022 | Comparison of duration of Kangaroo Mother Care (KMC) and its effects on infant and maternal stress | 112 infants born 28-34 weeks and their mothers, divided into two groups: Those who received an average ≥90 min KMC over the 12 study days vs those who received an average of <90 min per study day of KMC | Maternal and infant cortisol; EPDS |
| Welch MG, Halperin MS, et al | Depression and anxiety symptoms of mothers of preterm infants are decreased at 4 months corrected age with Family Nurture Intervention in the NICU | United States  2016 | Comparison of standard of care to standard of care plus the Family Nurture Intervention (FNI), a series of dyadic, multisensory calming activities | 115 mothers of 150 infants born 26-34 weeks gestation (control n=56; FNI = 59) | CES-D; STAI; BISBAS |
